# Evolution of Mitochondrially Derived Peptides Humanin and MOTSc, and Changes in Insulin Sensitivity during Early Gestation in Women with and without Gestational Diabetes

**DOI:** 10.3390/jcm11113003

**Published:** 2022-05-26

**Authors:** David Ruiz, Miguel Santibañez, Bernardo Alio Lavín, Ana Berja, Coral Montalban, Luis Alberto Vazquez

**Affiliations:** 1 Department of Endocrinology, Marqués de Valdecilla University Hospital, Avda Valdecilla, 39008 Santander, Spain; coral.montalban@scsalud.es (C.M.); luisvazquezsalvi@gmail.com (L.A.V.); 2 Nursing Department, University of Cantabria-IDIVAL, Avda Valdecilla, 39008 Santander, Spain; miguel.santibanez@unican.es; 3 Department of Clinical Analysis, Marqués de Valdecilla University Hospital, Avda Valdecilla, 39008 Santander, Spain; bernardoalio.lavin@scsalud.es (B.A.L.); ana.berja@scsalud.es (A.B.); 4 Department of Medicine and Psychiatry, University of Cantabria, Avda Valdecilla, 39008 Santander, Spain

**Keywords:** mitochondria-derived peptides, humanin, MOTSc, gestational diabetes mellitus, HOMA-IR, insulin resistance

## Abstract

Our purpose is to study the evolution of mitochondrially derived peptides (MDPs) and their relationship with changes in insulin sensitivity from the early stages of pregnancy in a cohort of pregnant women with and without gestational diabetes (GDM). MDPs (humanin and MOTSc) were assessed in the first and second trimesters of gestation in 28 pregnant women with gestational diabetes mellitus (GDM) and a subgroup of 45 pregnant women without GDM matched by BMI, age, previous gestations, and time of sampling. Insulin resistance (IR) was defined as a HOMA-IR index ≥70th percentile. We observed a significant reduction in both humanin and MOTSc levels from the first to the second trimesters of pregnancy. After adjusting for predefined variables, including BMI, statistically nonsignificant associations between lower levels of humanin and the occurrence of a high HOMA-IR index were obtained (adjusted OR = 2.63 and 3.14 for the first and second trimesters, linear *p*-trend 0.260 and 0.175, respectively). Regarding MOTSc, an association was found only for the second trimester: adjusted OR = 7.68 (95% CI 1.49–39.67), linear *p*-trend = 0.012. No significant associations were observed in humanin change with insulin resistance throughout pregnancy, but changes in MOTSc levels were significantly associated with HOMA-IR index: adjusted OR 3.73 (95% CI 1.03–13.50). In conclusion, MOTSc levels, especially a strong decrease from the first to second trimester of gestation, may be involved in increasing insulin resistance during early gestation.

## 1. Introduction

Gestational diabetes mellitus (GDM) is one of the most important complications associated with pregnancy. Its onset is related to defects in insulin secretion, insulin sensitivity, or a combination of both, and these conditions may become apparent early in pregnancy [1]. However, international guidelines recommend screening for GDM only late in the second trimester [2,3,4]. Despite this recommendation, nearly one-third of GDM diagnoses can occur before the 24th week, and these pregnant women with “early GDM” have an increased risk of complications compared to pregnant women with “late GDM” [5]. It is therefore essential to identify mediators that might play a role in the changes in insulin sensitivity from the early stages of gestation and to determine their predictive capacity for GDM occurrence.

Mitochondria are essential organelles for multiple aspects of cellular homeostasis. Mitochondrial dysfunction is implicated as a major contributing factor for a number of noncommunicable chronic diseases, including insulin resistance [6]. Moreover, impaired mitochondrial function may play a role in the risk of developing GDM; however, it is not known whether this dysfunction results in a primary defect in the pathophysiology of the disease [7]. Several previous studies that examined placental ultrastructure found significant dynamic [8] and structural [9] alterations in the mitochondria of pregnant women with GD. Mitochondria have their own circular genome (mitochondrial DNA, mtDNA) of approximately 16.5 Kilobases comprising 37 genes that encode 13 proteins of the respiratory chain, 22 tRNA, and 2 rRNA. However, in addition to their well-known function in cellular bioenergetics, different mitochondria-derived peptides (MDPs), which are small bioactive peptides encoded by short open reading frames (sORFs) in mtDNA, have been identified in the last few years [10]. To date, eight different MDPs have been described, acting as signaling agents in cytoprotection and energy regulation tasks [11]. Humanin, a 24 aa polypeptide encoded by the 16S rRNA coding region of mtDNA, has been associated with several homeostatic functions: cell survival factor [12], cytoprotection against oxidative stressors, activation of the chaperone-mediated autophagy pathway [13], and decreased apoptosis and protection from cell death by upregulation of mitochondrial glutathione (GSH), inhibition of ROS generation, and caspase 3 and 4 activation [14]. On the other hand, the mitochondrial open reading frame of 12S rRNA type-c (MOTSc) encoded by the 12S rDNA region of mtDNA is a 16 aa polypeptide expressed in various tissues and in circulation in rodents and humans, suggesting both a cell-autonomous and hormonal role [15]. Indeed, MOTSc promotes insulin sensitivity and beta-oxidation via AMPK [16] and directly regulates nuclear gene expression following nuclear translocation [17,18].

Collectively, these data support the hypothesis that humanin and MOTSc may be involved in changes in insulin sensitivity that arise from the early stages of pregnancy and the risk of gestational diabetes. Our aim is to describe, for the first time, the evolution of humanin and MOTSc during early pregnancy in women with and without GD and to analyze their relationship with the changes in insulin sensitivity that are triggered at this early stage of gestation.

## 2. Materials and Methods

### 2.1. Study Population

The study sample was drawn from a population included in a previous study designed to establish the reference thresholds of thyroid function parameters in the pregnant population of our geographical area. Recruitment occurred during 2016. The population was made up of healthy pregnant women who received care for their first pregnancy in the primary care clinics of area IV in Cantabria (Northern Spain). The criteria for inclusion were age ≥18 years, first visit within the first trimester of pregnancy, absence of thyroid functional disorders, and absence of chronic diseases (including diabetes). Exclusion criteria were having received fertility treatment and multiple gestations. All participants were invited to provide blood and urine samples in each of the gestation trimesters and to fill out a survey on sociodemographic aspects.

The initial sample included a total of 664 pregnant women. Forty-eight women were excluded because they experienced a miscarriage, ninety-three because they were found to have alterations in the parameters of thyroid function, and fifty-five because one of the samples from the first two trimesters was not available. Therefore, a total of 468 pregnant women without pregestational diabetes constituted our final study sample. We identified all pregnant women who were diagnosed with GDM in the second trimester, which resulted in a total of *n* = 40 (8.5%), and from the same study sample, we selected a subgroup of matched controls by BMI, age, previous gestations, and time of sampling (difference not exceeding three months). Finally, 12 pregnant women with GDM were excluded because we failed to identify matched controls, so the final study sample consisted of 28 pregnant women with GDM and 45 without GDM (controls). The number of controls was calculated to maintain a ratio greater than 1.5 controls for each GDM case [19,20].

### 2.2. Data Collection and Biomarkers

Through a structured questionnaire and review of medical records, information was collected on maternal age, weight in the first and second trimesters, height, obstetric history, and smoking habits. BMI in the first and second trimesters was calculated as weight in kilograms over height in meters squared. GDM was diagnosed according to the usual protocol of our health service. This protocol consists of a universal screening in two steps: a non-fasting oral overload test with 50 g of glucose in all pregnant women, and if the blood glucose value at the hour was ≥7.8 mmol/L, a diagnostic test was performed consisting of an oral glucose overload of 100 g with determination of fasting blood glucose and at 1, 2, and 3 h later. A positive result was defined as having two or more values above the thresholds established according to ADA criteria (NDDG): fasting ≥5.8 mmol/L; 1 h, ≥10.6 mmol/L; 2 h, ≥9.2 mmol/L; and 3 h, ≥8.0 mmol/L [21].

Blood samples were taken at 8:00 am while fasting between weeks 10 and 12 in the first trimester and between weeks 24 and 26 in the second. All samples were immediately centrifuged, and the resulting serum was frozen at −80 °C until analysis. For MDP analysis, serum samples were thawed at the same time in 2020. Humanin was evaluated by ELISA (Humanin MT-RNR2) (Cusabio Biotech Co., Ltd., Houston, TX, USA). The analytical sensitivity was 7 pg/mL, and no cross-reactions with humanin MT-RNR2 analogs were observed. The intra-assay reproducibility of the method was <8%, and the inter-assay reproducibility was <10%. The quantification of MOTSc was performed by ELISA (Cloud-Clone Corp, Katy, TX, USA). The analytical sensitivity was 0.97 ng/mL. Claims for specificity and absence of cross-reactivity were provided by the commercial company. The intra-assay reproducibility of the method was <10%, and the inter-assay reproducibility was <12%. Glucose was determined automatically by the glucose oxidase method in an Atellica CH analyzer (Siemens Healthcare Diagnostics, Inc., Newark, DE, USA). The analytical sensitivity was 6 mg/dL. The intra-assay reproducibility of the method was <1.6%, and the inter-assay reproducibility was <4.2%. Insulin was determined by an automated immunoassay in an Atellica IM analyzer (Siemens Healthcare Diagnostics, Inc, Newark, DE, USA). The analytical sensitivity was 0.3 mIUI/L. The intra-assay reproducibility of the method was <1.8% and inter-assay reproducibility was <3.6%. All assays were performed without knowledge of case–control status.

We used the calculation of the homeostasis model assessment (HOMA) to evaluate both insulin resistance (HOMA-IR), according to the formula fasting serum insulin (µU/mL) × fasting plasma glucose (mmol/L)/22.5, and beta cell function (HOMA-β), according to the formula 20 × fasting insulin (µU/mL)/fasting plasma glucose (mmol/L) − 3.5 in each trimester. We consider values ≥70th percentile (p70) as high HOMA-IR.

### 2.3. Statistical Analysis

Categorical variables are displayed as percentages, and the χ^2^ test was used for comparisons between groups. Continuous variables are summarized as the mean ± standard deviation (SD) or median (interquartile range), and comparisons between GDM cases and controls were performed using the Student’s t-test in cases of normal distribution or the Mann–Whitney U-test when appropriate. For intra-group first- and second-trimester comparisons, we used the Related-Samples *T* Test or Wilcoxon signed-rank test when appropriate. The correlation between humanin and MOTSc levels and age, BMI in each trimester, and HOMA-IR as continuous variables was estimated through Spearman’s correlation coefficient. To determine and compare the predictive capacity of each variable on the risk of a high HOMA-IR index or developing GDM, receiver operating characteristic (ROC) curves and the area under the curve (AUC) were determined.

We used a multivariate logistic regression analysis to calculate the strength of the associations. MDPs were dichotomously categorized (high versus low values) according to the median and odds ratios (ORs) with 95% confidence intervals (95% CIs) adjusted for smoking status, body mass index (BMI), and age for a HOMA-IR ≥p70 and adjusted for smoking status for GDM. In addition, when a significant association was identified, exposure–response trends (biological gradient, dose–response pattern) were estimated using a logistic regression model with all potential confounders and ordinal categorizing of the variables according to tertiles. The third tertile was the reference category except for kinetics, which is the change or decrease in each MDP value (related-sample difference between the first and second trimesters).

The statistical analysis was performed using SPSS statistical software package version 22.0 (SPSS Inc., Chicago, IL, USA). The level of statistical significance was set at 0.05, and all tests were two-tailed.

This study was conducted according to the guidelines of the Declaration of Helsinki and approved by the Clinical Research Ethics Committee of Cantabria (CEIC: 2020-428). Written informed consent was obtained from each subject.

## 3. Results

### 3.1. Basal Characteristics

The baseline characteristics of pregnant women are summarized in Table 1. The mean age was 32.7 ± 5.1 years, and the BMI was 25.2 ± 5.0 and 27.6 ± 4.7 kg/m^2^ in the first and second trimesters, respectively, with no significant differences between pregnant women with and without diabetes, except for a higher percentage of pregnant women who smoked in the GDM group: 25% vs. 6.7% (*p* = 0.038).

### 3.2. Humanin and MOTSc MDP Levels in the First and Second Trimesters

**Figure 1:**
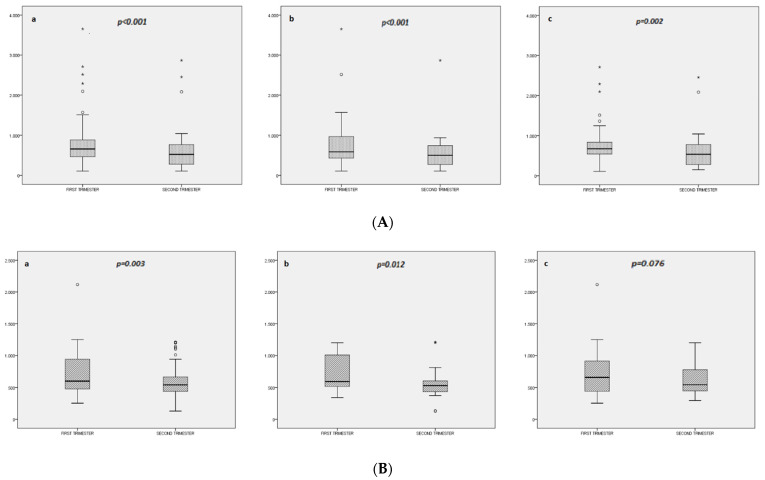
(**A**). Box plots of humanin levels in the first and second trimesters in the total cohort (**a**) and restricted to women with gestational diabetes mellitus (GDM) (**b**) and controls (non-GDM) (**c**). (**B**). Box plots of MOTSc levels in the first and second trimesters in the total cohort (**a**) and restricted to women with gestational diabetes mellitus (GDM) (**b**) and controls (non-GDM) (**c**). The box plots represent lines, boxes represent the median and interquartile range, and whiskers calculate outlier data. The Wilcoxon signed-rank test was used for the paired-samples comparisons between trimesters. * and ° represent outliers values.

In the total cohort, we found a significant decrease in humanin and MOTSc levels from the first to second trimester of gestation (Table 2). Humanin decreased significantly from the first to second trimester of gestation in both groups of pregnant women (GDM group and non-GDM group) (Figure 1A). However, MOTSc decreased significantly in only the GDM group (*p* = 0.012), while the decrease was not significant in the non-GDM group (*p* = 0.076) (Figure 1B). In one pregnant woman, humanin levels were abnormally high in the second trimester. The result was repeated and remained abnormally high. The results obtained by excluding this subject in a sensitivity analysis showed less difference in the comparison of kinetics between groups with no other significant differences. Supplementary Table S1 compares the humanin or MOTSc levels between pregnant women with and without GDM for each trimester of pregnancy separately.

### 3.3. Association with Insulin Resistance

**Figure 2:**
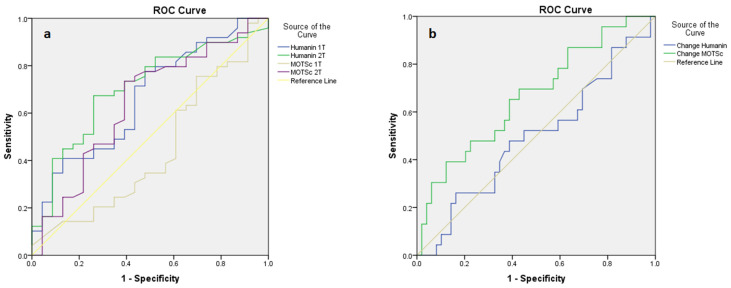
Receiver operating characteristic (ROC) curve for high HOMA-IR in relation to mitochondria-derived peptides in the first trimester (1T) and second trimester (2T) separately (**a**) and in relation to sample-related changes for each peptide throughout pregnancy (**b**). (**a**) High HOMA-IR was defined as a value ≥70th percentile (≥1.90 1T and ≥2.28 2T); Humanin 1T: AUC 0.698 (95% CI 0.569–0.826); Humanin 2T: AUC 0.705 (95% CI 0.578–0.831); MOTSc 1T: AUC 0.497 (95% CI 0.355–0.638); MOTSc 2T. AUC: 0.640 (95% CI 0.498–0.782). (**b**) High HOMA-IR was defined as a value ≥70th percentile (≥1.90 1T and ≥2.28 2T); Change in Humanin: AUC 0.494 (95% CI 0.348–0.640); Change in MOTSc: AUC 0.665 (95% CI 0.531–0.800).

AUC values ≤0.705 for both humanin and MOTSc peptides were obtained in the first and second trimesters separately for the risk of a high HOMA-IR index (≥p70), as well as for the sample-related change for each peptide throughout pregnancy (Figure 2).

We found a statistically significant crude association between low levels of humanin (below median) and a higher risk of presenting a high HOMA-IR index (≥p70) (Supplementary Table S2) in both the first and second trimesters of gestation, with a statistically significant linear *p*-trend when ordinal categorizing according to tertiles: OR for the lowest values of humanin in first trimester = 7.22 (95% CI 1.70–30.64), linear *p*-trend = 0.006; OR for the lowest values of humanin in second trimester = 7.00 (95% CI 1.67–29.35), linear *p*-trend = 0.006 (Table 3). We identified a negative correlation between BMI and humanin in both the first (r value −0.343; *p* = 0.003) and second trimesters (r value −0.358; *p*=0.006) (data not shown in tables). In this sense, after including BMI in the multivariate regression model in addition to maternal age and smoking habit, the association with HOMA-IR decreased in strength and lost statistical significance: adjusted OR for the lowest values of humanin = 2.63 and 3.14, linear *p*-trend = 0.260 and 0.175 for the first and second trimesters, respectively (Table 3).

Regarding MOTSc, no association was identified between its levels and a high HOMA-IR index in the first trimester (Supplementary Tables S2 and S3). However, in the second trimester, an association between low MOTSc levels and a higher risk of elevated HOMA-IR index was observed: adjusted OR for the lowest values of MOTSc = 7.68 (95% CI 1.49–39.67), linear *p*-trend = 0.012 (Table 3).

Regarding the evolution of MDPs throughout pregnancy, a positive correlation between a greater decrease in MOTSc levels throughout pregnancy and higher HOMA-IR in the second trimester of pregnancy was observed (r value 0.262; *p* = 0.026) (Supplementary Table S3). After adjusting the results for BMI, age, and smoking status, pregnant women with a higher decrease in MOTSc levels presented a higher risk of elevated HOMA-IR index: adjusted OR 3.73 95% CI 1.03–13.50 (*p* = 0.045) (Supplementary Table S4). No significant crude or adjusted associations were observed in relation to the change in humanin levels (Supplementary Table S4).

### 3.4. Association with GDM

Changes in MOTSc levels showed higher AUC levels among those analyzed in relation to the risk of developing GDM: AUC 0.576 (95% CI 0.442–0.710) (Supplementary Figure S1).

In the regression analysis, in agreement with the ROC approach, all the assessed parameters showed positive associations with the risk of gestational diabetes (adjusted OR >1), but they were of a small magnitude and did not reach statistical significance (Supplementary Table S5).

## 4. Discussion

We observed a significant decrease between the first and second trimesters of gestation in both humanin and MOTSc levels. The decrease in humanin was comparable among pregnant women who later developed GDM and those who did not. However, the decrease in MOTSc was only significant in the group of pregnant women who developed diabetes.

To our knowledge, there are no published studies on the plasma levels of MDPs (humanin and MOTSc) in the early stage of gestation and their evolution throughout pregnancy. Furthermore, only one previous study has evaluated humanin levels during pregnancy with a single determination between 24 and 28 weeks [22]. In contrast with our results, in the study of Ma. Y. et al., humanin levels were significantly lower in women with GD than in controls. It is possible that these differences may be largely explained by significant differences in weight between the GD group and the non-GD group at the time of humanin sampling. Another study identified higher levels of MOTSc in obese versus non-obese pregnant women [23].

We also explored the relationship between MDPs and insulin resistance during early gestation. We found an association between low levels of humanin and a higher risk of presenting a high HOMA-IR index (≥p70) in both the first and second trimesters of gestation; however, this association decreased in strength and lost statistical significance after adjusting the analysis for BMI. In fact, the identification of a negative correlation between the levels of humanin and BMI in both trimesters reinforces the hypothesis that BMI is an important confounder when interpreting the relationship between humanin and insulin sensitivity.

Regarding MOTSc, surprisingly, the findings were not the same in the first trimester, where we did not identify a significant association with the HOMA-IR index, and the second trimester, where we observed a significant relationship between the MOTSc levels and the HOMA-IR index with a higher risk of presenting a HOMA-IR index ≥p70 in pregnant women with lower MOTSc levels. This association was maintained after adjusting the analysis for both BMI and age, and it was mainly derived from a higher risk among pregnant women with MOTSc levels in the lower tertile. In addition, a more pronounced decline in MOTSc levels between the first and second trimesters resulted in a higher risk of having a HOMA-IR index ≥p70. In our sample, a disparity in the number of smokers was observed between the GDM group (7/28 were smokers) and the control group, where only 3 were smokers. As smoking could be a stressor altering MOTSc levels, also being associated with the development of insulin resistance, we included it as a confounding variable in the multivariate model. Additionally, we performed a sensitivity analysis excluding *n* = 10 smokers, where MOTSc results were maintained. Therefore, our results suggest that MOTSc levels, especially the decrease between the first and second trimesters of gestation, are associated with an increased risk of insulin resistance during early gestation. The correlation with the value of HOMA-IR in the second trimester, statistically significant although small, as well as the associations found when dichotomizing insulin resistance based on a cutoff point ≥p70 of HOMA-IR, would support this hypothesis.

Our findings are consistent with previous studies that have identified a relationship between MDPs and insulin resistance. There is evidence of the insulin-sensitizing properties of MDPs, mainly derived from cellular and animal models [11]. MOTSc is detected in the circulation, and its target organs are primarily skeletal muscle and fat. Administration of MOTSc in mice resulted in increased glucose uptake, primarily by skeletal muscle tissue, prevented the development of insulin resistance induced by a high-fat diet, and reversed age-associated insulin resistance via activation of AMPK and SIRT1 [16]. Furthermore, MOTSc improves insulin sensitivity and increases beta-oxidation by targeting three metabolic pathways: sphingolipid metabolism, monoacylglycerol metabolism, and dicarboxylate metabolism [24]. Humanin has been shown to decrease beta cell apoptosis in vitro and delay the development of diabetes in mouse NOD in vivo [25]. Finally, it has been reported that people with type 2 diabetes mellitus have lower levels of humanin and MOTSc than people without diabetes, and their levels correlate with the HbA1c value [26].

Despite the relationship of MDPs with insulin sensitivity during pregnancy, their predictive capacity for the development of GDM was poor according to the AUC values obtained. This probably reflects the complexity of GDM, where insulin resistance is only one of the factors involved in its physiopathology. In fact, the ability to diagnose GDM in the first trimester remains controversial, and all parameters investigated have been poorly predictive of oral glucose tolerance test outcomes in the third trimester [27]. The highest precision achieved by a model, defined as the summation of seven binary variables recommended by the National Institutes of Health (NIH), was only 30%, and its AUC for GDM was 0.682 [28].

Our study has several limitations. First, the small sample size could attenuate our ability to identify significant differences between pregnant women with and without GDM. Second is the single-center nature of the study, and third is the use of HOMA-IR as a marker of sensitivity to insulin. However, we speculate that this mathematical model may be suitable to estimate the longitudinal changes in insulin sensitivity in our study population and has been shown to be an independent risk factor for the development of GDM [29]. Furthermore, a good correlation between HOMA-estimated insulin resistance and the euglycemic clamp [30] or minimal model [31] has been described. Further studies with larger sample sizes and, given the possibility of cross-reactivity with current commercial kits, using different methodologies (other ELISAs with in situ specificity tests, or ELISAs contrasted by mass spectrometry) should extend and corroborate our results. In relation to the strengths of the study, it is worth mentioning the effort to study the associations through different analysis strategies, while also exploring the dose–response pattern, and the control of confounding in the design phase through matching and using multivariate analysis.

## 5. Conclusions

We found a significant decline in humanin and MOTSc levels between the first and second trimesters of pregnancy. The decrease in humanin was significant in pregnant women who developed diabetes and those who did not, while that of MOTSc was only significant in pregnant women who developed diabetes. Moreover, a greater decrease in MOTSc levels is associated with a higher risk of presenting a high HOMA-IR in the second trimester, while the relationship between humanin and HOMA-IR is attenuated and becomes nonsignificant after including BMI in the analysis. Thus, our results suggest that MOTSc levels, especially a strong decrease between the first and second trimesters of gestation, may be involved in the progressive increase in insulin resistance starting from early gestation.

## Figures and Tables

**Table 1:** Characteristics of the study participants presented as the total study population and stratified according to gestational diabetes mellitus (GDM) status.

Variable	Total	GDM	Non-GDM	*p* Value
	*(n = 73)*	*(n = 28)*	*(n = 45)*	
*Age, (yr) (mean ± SD)*	32.7 ± 5.1	32.8 ± 5.4	32.6 ± 4.9	*0.937*
*BMI (kg/m^2^) (mean ± SD)*				
First Trimester	25.2 ± 5.0	25.4 ± 5.6	25.0 ± 4.7	*0.851*
Second Trimester	27.6 ± 4.7	27.9 ± 5.0	27.4 ± 4.6	*0.704*
*Race/ethnicity*				
Non-Hispanic white	70 (95.8%)	28 (100%)	42 (93.3%)	*0.565*
African	1 (1.3%)		1 (2.2%)	
Hispanic	2 (2.7%)		2 (4.4%)	
*Previous pregnancies*				
None, *n* (%)	28 (38.4%)	10 (35.7%)	18 (40.0%)	*0.935*
1, *n* (%)	30 (41.1%)	12 (42.9%)	18 (40.0%)	
+1, *n* (%)	15 (20.5%)	6 (21.4%)	9 (20.0%)	
*Tobacco (yes)*	10 (13.7%)	7 (25.0%)	3 (6.7%)	*0.038*
*Gestational age (weeks) (mean ± SD)*				
First Trimester	10.37 ± 0.77	10.42 ± 0.57	10.33 ± 0.87	*0.262*
Second Trimester	25.09 ± 1.45	25.17 ± 1.38	25.04 ± 1.50	*0.623*

Comparisons between groups were performed by χ^2^ (categorical variables) or Student’s *t*-test if normally distributed or the Mann–Whitney U-test if non-normally distributed (continuous variables).

**Table 2:** Evolution of mitochondria-derived peptides HOMA-IR and HOMA-β between the first and second trimesters of gestation in the total cohort.

	First Trimester	Second Trimester	*p* Value
**Humanin (pg/mL), mean (SD)**	797.9 ± 607.7	697.2 ± 523.0	*<0.001*
**MOTSc (ng/mL), mean (SD)**	725.1 ± 332.8	592.0 ± 250.5	*0.003*
**Glucose (mmol/L), mean (SD)**	4.3 ± 0.3	4.4 ± 0.6	*0.400*
**Insulin (µU/mL), mean (SD)**	8.2 ± 4.5	11.1 ± 10.1	*0.001*
**HOMA-IR, mean (SD)**	1.6 ± 0.9	2.3 ± 2.5	*0.006*
**HOMA-β (%), mean (SD)**	223.2 ± 144.1	288.1 ± 262.0	*0.039*

MDPs: Mitochondria-derived peptides. Related-sample comparisons across trimesters were performed by the Wilcoxon signed-rank test.

**Table 3:** Association between levels of mitochondria-derived peptides and the HOMA-IR index during the first and second trimesters of gestation.

		HOMA-IR Low	HOMA-IR High						
MDPs	Cutoff Points	*n* = 48 (1T) *n* = 49 (2T)	*n* = 24 (1T) *n*= 23 (2T)	Crude OR	(95%	CI)	aOR ^a^	(95%	CI)
**First trimester Humanin (pg/mL) (Tertiles)**									
High (reference)	790+	20	3	1.00	--		1.00	--	
Medium	567–789	16	8	3.33	0.76	14.65	2.22	0.45	10.98
Low	≤566	12	13	7.22	1.70	30.64	2.63	0.51	13.43
*p* linear trend				*0.006*			*0.260*		
**First trimester MOTSc (ng/mL) (Tertiles)**									
High (reference)	823.3+	16	7	1.00	--		1.00	--	
Medium	526.9–823.2	15	10	1.52	0.46	5.04	1.23	0.29	5.10
Low	≤526.8	17	7	0.94	0.27	3.29	1.02	0.24	4.23
*p* linear trend				*0.918*			*0.992*		
**Second trimester Humanin (pg/mL) (Tertiles)**									
High (reference)	648+	21	3	1.00	--		1.00	--	
Medium	374–647	15	7	3.27	0.72	14.73	3.18	0.60	16.78
Low	≤373	13	13	7.00	1.67	29.35	3.14	0.64	15.44
*p* linear trend				*0.006*			*0.175*		
**Second trimester MOTSc (ng/mL) (Tertiles)**									
High (reference)	586.9+	19	5	1.00	--		1.00	--	
Medium	477.1–586.8	18	5	1.06	0.26	4.27	1.43	0.26	7.84
Low	≤477.0	12	13	4.12	1.17	14.50	7.68	1.49	39.67
*p* linear trend				*0.022*			*0.012*		

Note: One case was excluded because of missing insulin values in the first trimester, and one control was excluded because of missing glucose values in the second trimester. High HOMA-IR was considered ≥70th percentile of its distribution (≥1.90 1T and ≥2.28 2T). ^a^ aOR = odds ratios adjusted for maternal age, BMI, and smoking habit.

## Data Availability

The datasets generated during and/or analyzed during the current study are not publicly available but are available from the corresponding author upon reasonable request.

## Supplementary Materials

The following supporting information can be downloaded at: https://www.mdpi.com/article/10.3390/jcm11113003/s1, Table S1: Mitochondria-derived peptides (MDPs) in the first and second trimesters of gestation in GDM (cases) and controls; Table S2: Crude and adjusted odds ratio and 95% CI according to median mitochondria-derived peptides on the risk of high HOMA-IR in the first (1T) and second trimesters (2T) of gestation; Table S3: Spearman rank correlation bivariate analysis of variables associated with HOMA-IR in the first and second trimesters of gestation separately; Table S4: Association between changes in levels of mitochondria-derived peptides throughout pregnancy and the HOMA-IR index; Table S5: Association between changes in levels of mitochondria-derived peptides throughout pregnancy and the occurrence of gestational diabetes mellitus (GDM); Figure S1: Receiver operating characteristic (ROC) curve for mitochondria-derived peptides (MDPs): (a) in the first (1T) and second trimesters (2T), and (b) changes across gestation built on gestational diabetes mellitus (GDM).
