# Supplementary material for: Evolution of Mitochondrially Derived Peptides Humanin and MOTSc, and Changes in Insulin Sensitivity during Early Gestation in Women with and without Gestational Diabetes
Source: J Clin Med. 2022 May 26;11(11):3003. doi: 10.3390/jcm11113003 (PMC9181699; doi:10.3390/jcm11113003)
Supplement: Supplementary file 1 [file jcm-11-03003-s001.zip › Supplementary material Rv.pdf]

Evolution of mitochondrial-derived peptides, humanin and MOTSc and changes in insulin sensitivity during early gestation

Supplementary material

Supplementary files

Supplementary Table S1. Mitochondrial-derived peptides (MDPs) in the first and second trimesters of gestation in GDM (cases) and controls

Variables	*Total* (n= 73)	*GDM* (n= 28)	*Controls* (n=45)	*p value*
First trimester Humanin (pg/mL), mean (SD)	797.9±607.7	817.4±740.9	785.8±516.7	*0.507*
First trimester Humanin (pg/mL), median (IQR)	658.0 (455.0-905.0)	586.0 (422.5-980.0)	672.00 (515.0-859.0)	
Second trimester Humanin (pg/ml), mean (SD)	697.2±523.0	593.3±501.0	761.9±1205.9	*0.910*
Second trimester Humanin (pg/ml), median (IQR)	523.0 (276.0-771.0)	501.0 (269.5-754.0)	536.0 (276.5-798.0)	
First trimester MOTSc (ng/mL), mean (SD)	725.1±332.8	727.5±292.8	723±358.6	*0.798*
First trimester MOTSc (ng/mL), median (IQR)	600.0 (473.7-973.2)	592.8 (513.5-1008.7)	658.8 (436.5-919.2)	
Second trimester MOTSc (ng/ml), mean (SD)	592.0±250.5	554.2±236.5	615.5±258.7	*0.571*
Second trimester MOTSc (ng/ml), median (IQR)	541.5 (435.1-673.8)	530.2 (430.6-607.5)	543.6 (439.8-784.8)	
First trimester Glucose (mmol/l), mean (SD)	4.3±0.3	4.2±0.3	4.4±0.3	*0.135*
First trimester Glucose (mmol/l), median (IQR)	4.3 (4.0-4.6)	4.3 (4.0-4.5)	4.3 (4.1-4.6)	
Second trimester Glucose (mmol/l), mean (SD)	4.4±0.6	4.6±0.8	4.3±0.4	*0.181*
Second trimester Glucose (mmol/l) median (IQR)	4.4 (4.0-4.6)	4.4 (4.1-4.8)	4.3 (4.0-4.5)	
First trimester Insulin (µU/mL), mean (SD)	8.2±4.5	7.7±3.9	8.5±4.8	*0.457*
First trimester Insulin (µU/mL), median (IQR)	7.3 (4.7-10.6)	6.3 (4.4-10.9)	7.6 (5.3-9.9)	
Second trimester Insulin (µU/mL), mean (SD)	11.1±10.1	11.1±6.9	11.0±11.7	*0.266*
Second trimester Insulin (µU/mL), median (IQR)	9.4 (6.6-11.7)	9.8 (8.0-12.1)	9.0 (5.9-11.2)	
First trimester HOMA-IR, mean (SD)	1.6±0.9	1.5±0.8	1.7±1.0	*0.380*
First trimester HOMA-IR, median (IQR)	1.3 (0.9-2.0)	1.2 (0.7-2.0)	1.5 (0.9-2.0)	
Second trimester HOMA-IR, mean (SD)	2.3±2.5	2.5±2.7	2.1±2.4	*0.166*
Second trimester HOMA-IR, median (IQR)	1.8 (1.2-2.3)	1.8 (1.5-2.5)	1.7 (1.0-2.2)	
First trimester HOMA-β (%), mean (SD)	223.2±144.1	234.5±151.9	216.5±140.6	*0.646*
First trimester HOMA-β (%), median (IQR)	176.2 (125.1-277.9)	229.3 (113.4-278.2)	173.2 (126.2-278.8)	
Second trimester HOMA-β (%), mean (SD)	288.1±262.0	277.4±265.1	295.0±262.8	*0.432*
Second trimester HOMA-β (%), median (IQR)	208.0 (146.7-356.2)	195.0 (146.7-258.9)	220.0 (147.8-360.1)	

GDM: Gestational diabetes mellitus. Comparisons across groups were performed by the Mann-Whitney U-test.

Supplementary Table S2. Crude and adjusted odds ratio and 95% CI according to median mitochondrial-derived peptides on the risk of high HOMA-IR in the first (1T) and second trimesters (2T) of gestation.

		HOMA-IR low	HOMA-IR high						
Biomarkers	*Cut-of points*	*N=48 (1T) N=49 (2T)*	*N=24 (1T) N=23 (2T)*	*Crude OR*	*(95%*	*CI)*	*aOR[a]*	*(95%*	*CI)*
First trimester Humanin (pg/mL) (Median)									
High (reference)	659+	27	8	1.00			1.00		
Low	≤ 658	21	16	2.57	0.92	7.15	1.85	0.57	5.98
First trimester MOTSc (ng/ml) (Median)									
High (reference)	600.1+	25	11	1.00			1.00		
Low	≤ 600.0	23	13	1.28	0.48	3.43	2.47	0.72	8.52
Second rimester Humanin (pg/mL) (Median)									
High (reference)	524+	30	6	1.00			1.00		
Low	≤ 523	19	17	4.47	1.50	13.36	2.12	0.60	7.46
Second trimester MOTSc (ng/ml) (Median)									
High (reference)	541.6+	27	8	1.00			1.00		
Low	≤ 541.5	22	15	2.30	0.82	6.42	3.82	1.02	14.24

Note: One case was excluded because of missing insulin values in the first trimester, and one control was excluded because of missing glucose values in the second trimester.

High HOMA-IR was considered ≥ 70th percentile of its distribution (≥ 2.28).

[a]aOR= Odds ratios adjusted for maternal age, BMI and smoking habit.

Supplementary Table S3. Spearman rank correlation bivariate analysis of variables associated with HOMA-IR in the first and second trimesters of gestation separately.

	First Trimester		*Second Trimester*	
	r	***p***	***r***	***p value***
BMI	0.513**	<0.0001	0.433**	*0.0001*
Age	0.009	0.942	0.179	*0.133*
Humanin	-0.324**	0.005	0.186	*0.118*
MOTSc	0.037	0.759	-0.086	*0.471*
Humanin Change	0.189	0.113	-0.059	*0.622*
MOTSc Change	0.165	0.166	0.262*	*0.026*

*2-tailed correlation significance, p<0.05. ** 2-tailed correlation significance, p<0.01.

Supplementary table S4. Association between changes in levels of mitochondrial-derived peptides throughout pregnancy and the HOMA-IR index.

		**HOMA-IR low**	**HOMA-IR high**						
MDPs Change	***Cutoff points***	***N=49***	***N=23***	***Crude OR***	***(95%***	***CI)***	***aOR[a]***	***(95%***	***CI)***
Change in Humanin (pg/mL)									
Low (reference)	≤ 122.00	25	11	1.00			1.00		
High (above median)	123.00+	24	12	1.14	0.42	3.06	1.71	0.51	5.73
Change in MOTSc (pg/mL)									
Low (reference)	≤ 52.80	28	8	1.00			1.00		
High (above median)	52.81+	21	15	2.50	0.89	6.99	3.73[b]	1.03	13.50

Note: One control was excluded because of missing glucose values in the second trimester.

High HOMA-IR was considered ≥ 70th percentile of its distribution (≥ 2.28).

[a] aOR= Odds ratios adjusted for maternal age, BMI and smoking habit.

[b] *p* value=0.045.

Supplementary table S5. Association between changes in levels of mitochondrial-derived peptides throughout pregnancy and the occurrence of gestational diabetes mellitus (GDM).

		**Controls**	**GDM**						
MDPs	***Cutoff points***	***N=45***	***N=28***	***Crude OR***	***(95%***	***CI)***	***aOR***[a]	***(95%***	***CI)***
First trimester Humanin (pg/mL) (Median)									
High (reference)	659+	24	12	1.00			1.00		
Low	≤ 658	21	16	1.52	0.59	3.94	1.44	0.54	3.85
First trimester MOTSc (ng/ml)(Median)									
High (reference)	600.1+	23	13	1.00			1.00		
Low	≤ 600.0	22	15	1.21	0.47	3.10	1.35	0.50	3.63
Second trimester Humanin (pg/mL) (Median)									
High (reference)	524+	23	13	1.00			1.00		
Low	≤ 523	22	15	1.21	0.47	3.10	1.23	0.46	3.28
Second trimester MOTSc (ng/ml)(Median)									
High (reference)	541.6+	23	13	1.00			1.00		
Low	≤ 541.5	22	15	1.21	0.47	3.10	1.23	0.46	3.28
Change in Humanin (pg/mL)									
Low (reference)	≤ 122.00	24	13	1.00			1.00		
High (above median)	123.00+	21	15	1.32	0.51	3.40	1.34	0.50	3.56
Change in MOTSc (pg/mL)									
Low (reference)	≤ 52.80	24	13	1.00			1.00		
High (above median)	52.81+	21	15	1.32	0.51	3.40	1.34	0.50	3.56

[a]aOR= Odds ratios adjusted for maternal age, BMI and smoking habit.

Supplementary Figure S1. Receiver operating characteristic (ROC) curve for mitochondrial-derived peptides (MDPs): (a) in the first (1T) and second trimesters (2T), and (b) changes across gestation built on gestational diabetes mellitus (GDM).

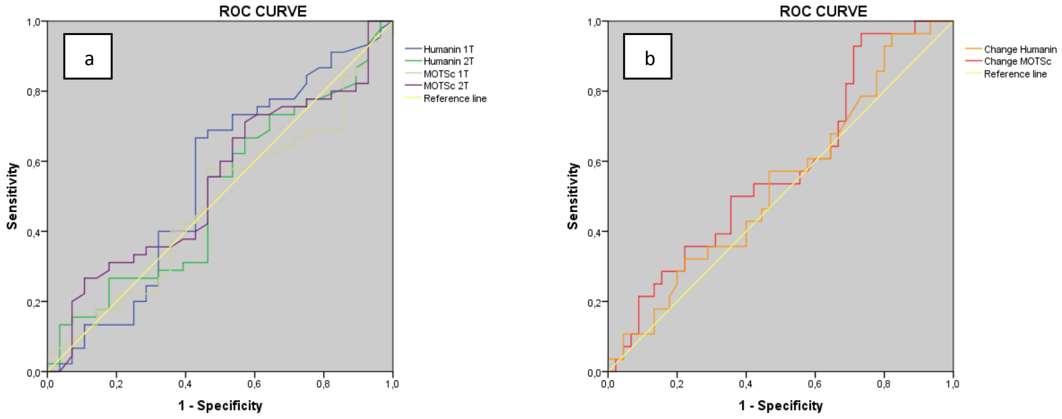

Humanin 1T: AUC 0.546; 95%CI (0.403-0,690);
Humanin 2T: AUC 0.508; 95%CI (0.370-0.646)
MOTSc 1T: AUC 0.482; 95%CI (0.346-0.618)
MOTSc 2T: AUC 0.540 (0.403-0.676)

Change Humanin: AUC 0.537; 95%CI (0.402-0.673)
Change MOTSc: AUC 0.576; 95%CI (0.442-0.710)
